# Occurrence of Fibrotic Tumor Vessels in Grade I Meningiomas Is Strongly Associated with Vessel Density, Expression of VEGF, PlGF, IGFBP-3 and Tumor Recurrence

**DOI:** 10.3390/cancers12103075

**Published:** 2020-10-21

**Authors:** Katharina Hess, Dorothee Cäcilia Spille, Alborz Adeli, Peter B. Sporns, Karina Zitta, Lars Hummitzsch, Julian Pfarr, Walter Stummer, Benjamin Brokinkel, Rouven Berndt, Martin Albrecht

**Affiliations:** 1 Vascular Research Center, University Hospital of Schleswig-Holstein, Campus Kiel, 24105 Kiel, Germany; 2 Department of Pathology, University Medical Center Schleswig-Holstein (UKSH), Campus Kiel, 24105 Kiel, Germany; 3 Department of Neurosurgery, University Hospital Münster, 48149 Münster, Germany; DorotheeCaecilia.Spille@ukmuenster.de (D.C.S.); Walter.Stummer@ukmuenster.de (W.S.); Benjamin.Brokinkel@ukmuenster.de (B.B.); 4 Institute of Clinical Radiology, University Hospital Münster, 48149 Münster, Germany; alborz.adeli@gmail.com (A.A.); Peter.Sporns@ukmuenster.de (P.B.S.); 5 Department of Anesthesiology and Intensive Care Medicine, University Hospital Schleswig-Holstein, Campus Kiel, 24105 Kiel, Germany; Karina.Zitta@uksh.de (K.Z.); Lars.Hummitzsch@uksh.de (L.H.); Martin.Albrecht@uksh.de (M.A.); 6 Department of Radiology and Neuroradiology, University Hospital of Schleswig-Holstein, Campus Kiel, 24105 Kiel, Germany; Julian.Pfarr@uksh.de; 7 Department of Cardiovascular Surgery, Vascular Research Center, University Hospital of Schleswig-Holstein, Campus Kiel, 24105 Kiel, Germany; Rouven.Berndt@uksh.de

**Keywords:** angiogenesis, antiangiogenic therapy, grade I meningioma, magnetic resonance imaging, recurrence, VEGF, PlGF, IGFBP-3

## Abstract

**Simple Summary:**

Meningiomas are one of the most common intracranial tumors and although up to 80% of meningiomas are classified as WHO grade I (benign), recurrence rates of up to 20% have been described. Surgical resection is the first-line therapy for meningiomas, but is not always possible depending on the location of the tumor. Therefore, the detection of predictive biomarkers and potential therapeutic targets is important for the postoperative care of meningioma patients. The analysis of tumor samples from a group of 297 meningioma patients demonstrated that histologically detected fibrotic tumor vessels (FTV) correlated with an increased risk of patient recurrence and with characteristic changes in radiological imaging. In addition, FTV were associated with increased vessel density and expression of VEGF, PlGF and IGFBP-3, which could serve as potential therapeutic markers.

**Abstract:**

Angiogenesis is a key feature during oncogenesis and remains a potential target of antiangiogenic therapy. While commonly described in high-grade lesions, vascularization and its correlation with prognosis in grade I meningiomas is largely unexplored. In the histological classification, not only the number but also the composition of blood vessels seems to be important. Therefore, tumor vessel density and fibrosis were correlated with clinical and imaging variables and prognosis in 295 patients with intracranial grade I meningioma. Expression of pro-angiogenic proteins within the meningiomas was investigated by proteome analyses and further validated by immunohistochemical staining. Fibrotic tumor vessels (FTV) were detected in 48% of all tumors and strongly correlated with vessel density, but not with the histopathological tumor subtype. Occurrence of FTV was correlated with a 2-fold increased risk of recurrence in both univariate and multivariate analyses. Explorative proteome analyses revealed upregulation of VEGF (vascular endothelial growth factor), PlGF (placental growth factor), and IGFBP-3 (insulin-like growth factor-binding protein-3) in tumors displaying FTV. Immunohistochemical analyses confirmed strong correlations between tumor vessel fibrosis and expression of VEGF, PlGF, and IGFBP-3. Presence of FTV was strongly associated with disruption of the arachnoid layer on preoperative MRI in univariate and multivariate analyses. In summary, the occurrence of fibrotic tumor vessels in grade I meningiomas is strongly associated with vessel density, disruption of the arachnoid layer, expression of VEGF, PlGF, IGFBP-3 and tumor recurrence.

## 1. Introduction

Meningiomas are the most common intracranial neoplasms and classified as WHO grade I in about 80% of all cases. Although generally considered benign, recurrences are common and have been described in up to 20% even after gross total resection. Malignant transformation was observed in 19.5% of patients with recurrent meningiomas [1,2,3,4]. Moreover, resection is often complicated in tumors in delicate anatomical locations. Correspondingly, adjuvant irradiation is recommended after subtotal resection, while chemotherapeutical options are sparsely available [5]. In recent years new data concerning the molecular pathological background of meningiomas have been obtained. This not only facilitates the diagnosis of meningiomas but also offers the possibility to identify new prognostic and therapeutic targets [6,7,8,9].

Several retrospective studies reported improved local tumor control following treatment with anti-angiogenic agents, such as bevacizumab, vatalanib, or sunitinib in recurrent, radioresistant, high-grade meningiomas [5,10,11,12]. The antiangiogenic effects of these agents are mainly based on blocking the vascular endothelial growth factor (VEGF) signaling pathway, one of the key mechanisms of tumorigenesis in general [13] and in the development of meningiomas in particular [14,15,16].

While studies about neovascularization as an indicator for angiogenesis and effects of anti-angiogenic therapies are usually restricted to high-grade meningiomas, investigations of grade I tumors are sparse. It appears reasonable that an increased tumor vascularization might correspond to a higher metabolic demand in biological aggressive lesions displaying more rapid tumor growth. An increased vessel density has also been described in a portion of benign meningiomas [16] and was shown to correlate with an augmented preoperative tumor volume [17]. Hence, further elucidation of the role of angiogenesis in grade I meningiomas might help to estimate the risk for tumor recurrence and the potential of anti-angiogenic drugs in these tumors. However, not only the number but also the composition of the blood vessels is important for the classification of meningiomas [18,19].

In this study, we therefore investigated vascular architecture and performed proteome profiling analyses and immunohistochemical stainings in a series of grade I meningiomas to explore possible morphological correlations with clinical and radiological variables.

## 2. Results

Two-hundred and ninety-five patients who underwent surgery for histopathologically confirmed grade I intracranial meningiomas in a single neurosurgical department between 1994 and 2015 were analyzed. Grading and diagnosis were performed according to the current 2016 WHO classification of brain tumors in all cases. Brain invasion was absent in all cases by definition. Clinical, histopathological, and radiological data are summarized in Table 1.

### 2.1. Vessel Density is Correlated with Fibrotic Tumor Vessels and Histopathological Tumor Subtypes

**Figure 1:**
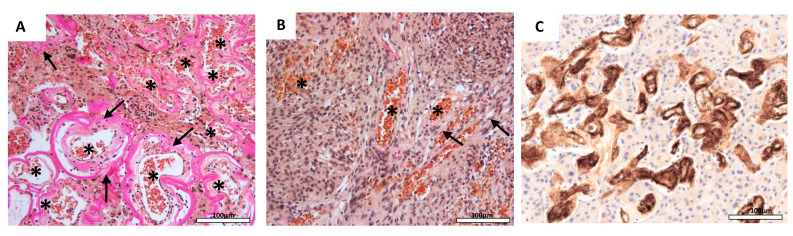
Vascular fibrosis and density in meningioma samples. Intratumoral blood vessels with thickened fibrotic vessel walls (**A**, arrow) and increased vessel density (**A**, asterisk) compared to inconspicuous blood vessels with narrow vessel walls (**B**, asterisk) and flat endothelium (**B**, arrow; Elastica–Van Gieson (EvG) staining, original magnification: 200×). Vessel density highlighted by anti-CD34 antibody (**C**; original magnification: 200×).

Histopathological analyses revealed fibrotic tumor vessels (FTV) in 140 of all 295 analyzed tumors (48%; Figure 1A). Fibrotic tumor vessels (FTV) were diagnosed by the presence of collagen-rich, low-cellular lining of the vessels’ walls. The histological slides were independently evaluated by two pathologists in a blinded manner. In case of discrepant findings, the results were discussed and slides were re-examined together. The number of vessels (vessel density, VD) per four high power fields (HPF) was quantified by EvG staining (mean 53.8 ± 39.5 vessels/4 HPF) and verified by CD34 staining (mean 52.8 ± 39.8 vessels/4 HPF; Supplementary Figure S1) and strongly correlated with FTV (*p* < 0.001). Pre-tests revealed a high intra-observer and inter-observer reproducibility with a κ-value of 0.82 for observer 1 and a κ-value of 0.90 for observer 2. Thus, the inter-observer variability was 0.82 demonstrating a high consistency of histological assessment. Tumor vessel fibrosis was similarly distributed among the different histopathological subtypes of the tumors (*p* = 0.136). Median VD differed between the histological grade I tumor subtypes and was 138 (range: 50–205), 32 (range: 8–201), 46 (range: 4–205), and 49 (range: 7–167) in angiomatous, transitional, meningothelial, fibrous, and secretory meningiomas, respectively (*p* < 0.001).

### 2.2. Vessel Fibrosis is Correlated with Tumor Recurrence

No correlations were found between the patients’ age or sex and the detection of FTV (*p* = n.s., each). Rates of FTV were similar in initially diagnosed meningiomas and tumors operated due to recurrence (*p* = n.s., each, Table 2).

**Figure 2:**
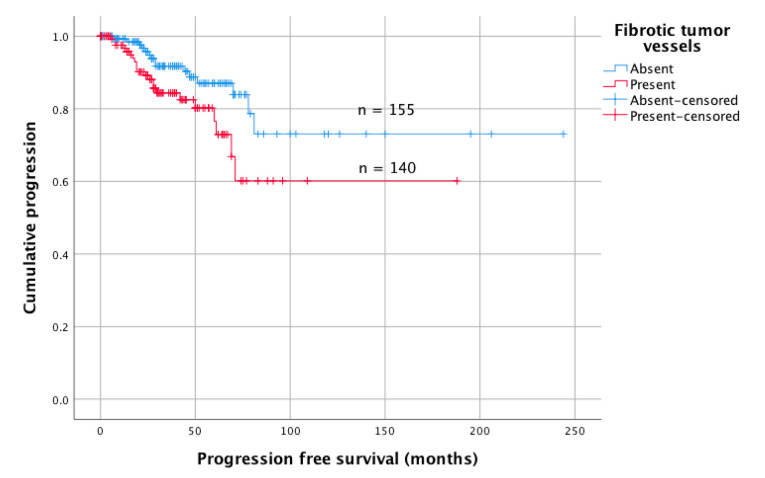
Kaplan–Meier showing correlations between fibrotic tumor vessels and prognosis. Progression-free survival was significantly shorter (*p* = 0.029, Log-rank test) in patients when intratumoral fibrotic tumor vessels were present (HR: 2.06, 95%CI 1.02–4.15; *p* = 0.043).

Data about tumor recurrence were available for 293 patients (99%). Within a median follow-up of 38 months, recurrence was observed in 37 patients (13%). Among all clinical and histopathological variables, univariate analyses revealed surgery for tumor recurrence/progression (HR: 4.74, 95%CI 2.06–10.87; *p* < 0.001), subtotal resection (HR: 2.28, 95%CI 1.16–4.47; *p* = 0.017), and the presence of FTV (HR: 2.05, 95%CI 1.06–3.98; *p* = 0.033, Figure 2), but not the VD (HR: 1.00, 95%CI 0.99–1.01) to be correlated with tumor progression. In multivariate analyses adjusted for patient´s age, sex, tumor location, indication for surgery, and the extent of resection, surgery for tumor relapse (HR: 5.52, 95%CI 2.21–13.81; *p* < 0.001) and FTV (HR: 2.06, 95%CI 1.02–4.15; *p* = 0.043) were identified as the only independent predictors for tumor recurrence.

### 2.3. Expression of Angiogenesis Related Proteins Correlates with Vessel Fibrosis and Vessel Density

**Figure 3:**
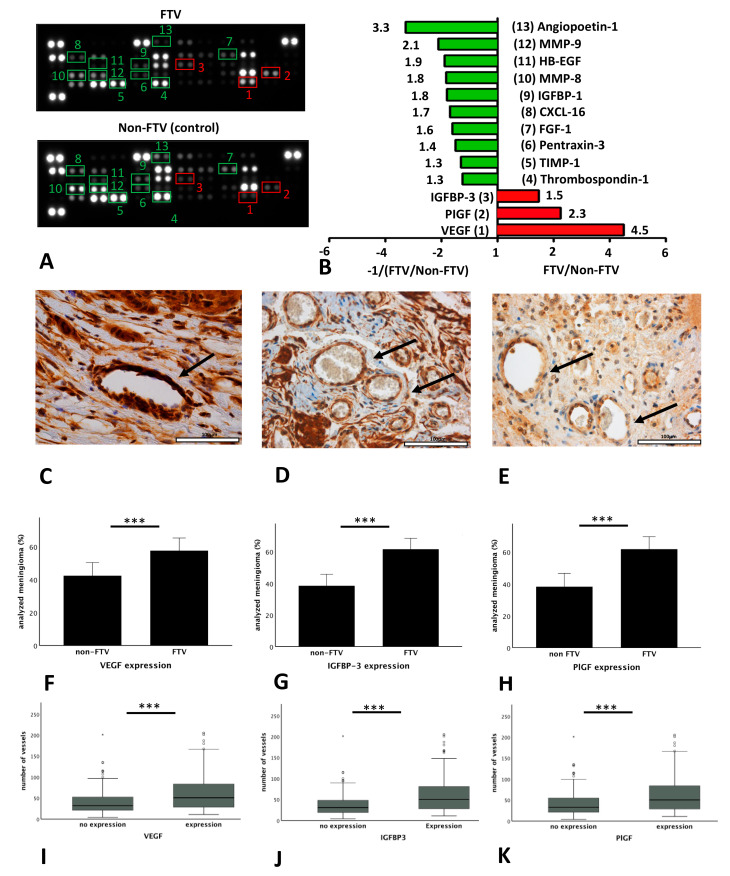
Expression of VEGF, IGFBP-3, and PlGF in meningioma vessels: Measurement of the optical density of meningioma samples with FTV and disruption of the arachnoid layer (FTV group, *n* = 8; A top) and without FTV and disruption of the arachnoid layer (non-FTV control group, *n* = 7; **A** bottom). The FTV group displays the distinct expression of various angiogenesis related proteins as compared to the non-FTV group (**B**). The immunohistochemical data confirm the results of the angiogenesis proteome profiler arrays and demonstrated significant differences in the endothelial expression (arrows) of vascular endothelial growth factor (VEGF; **C**), insulin-like growth factor-binding protein-3 (IGFBP-3; **D**), and placental growth factor (PlGF; **E**) evaluated using semi-quantitative analyses (**F**–**H**). Furthermore, the expression of VEGF, IGFBP-3, and PlGF correlated significantly with vessel density in the meningiomas (**I**–**K**). Original magnification: 400×. Error bars: 95%CI. *** *p* < 0.001.

Expression of angiogenesis related proteins was compared in tissue lysates of samples from patients with FTV (*n* = 8, FTV group) and without FTV (*n* = 7, non-FTV, control group) using angiogenesis proteome profiler arrays. Tumors were histologically classified as transitional (*n* = 5) and meningothelial meningiomas (*n* = 10). Expression of various angiogenesis-related proteins distinctly differed between both groups (Figure 3A,B). In detail, samples from the FTV group showed upregulation of VEGF (4.5-fold), PlGF (2.3-fold), and IGFBP-3 (1.5-fold). In contrast, angiopoetin-1 (3.3-fold), MMP-9 (matrix metalloproteinase-9; 2.1-fold), HB-EGF (heparin-binding endothelial growth factor-like growth factor; 1.9-fold), MMP-8 (matrix metalloproteinase-8; 1.8-fold), IGFBP-1 (insulin-like growth factor-binding protein-1; 1.8-fold), CXCL-16 (chemokine C-X-C motif ligand 16; 1.7-fold), FGF-1 (fibroblast growth factor-1; 1.6-fold), PTX3 (Pentraxin-3; 1.4-fold), TIMP-1 (tissue inhibitor of metalloproteinases-1; 1.3-fold), and thrombospondin-1 (1.3-fold) displayed lower expression in the FTV group as compared to the non-FTV (control) group.

Subsequently, expression of the three angiogenesis related proteins found to be upregulated in patients with FTV (VEGF, IGFBP-3, and PlGF) was semiquantitatively analyzed in the entire cohort by immunohistochemical staining (Figure 3C–E). Staining was successful in 286 of 295 samples (97%). Among those, target proteins were highly expressed in the endomembrane system of blood vessels. In fact, endothelial VEGF, IGFBP-3, and PlGF expression was found in 52% (*n* = 151), 61% (*n* = 177), and 47% (*n* = 136) of meningiomas, which differed significantly between meningiomas with FTV (58% (*n* = 87); 62% (*n* = 109); 62% (*n* = 84)); and without FTV (42% (*n* = 64); and 38% (*n* = 68) and 38% (*n* = 52); *p* < 0.001, respectively; Figure 3F–H). Similarly, VD was strongly correlated with VEGF (*p* < 0.001), IGFBP-3 (*p* < 0.001), and PlGF expression (*p* < 0.001; Figure 3I–K).

### 2.4. Vessel Fibrosis is Associated with Morphological Characteristics on MRI

**Figure 4:**
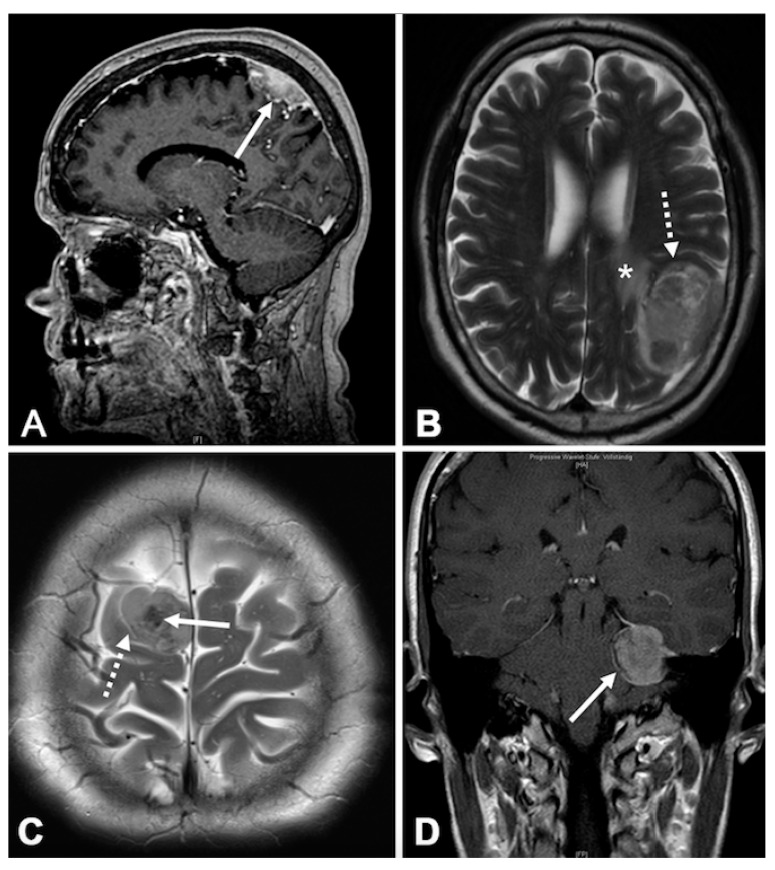
Illustrative samples of the analyzed characteristics on magnetic resonance imaging. Sagittal T1-weighted imaging shows an irregular, lobulated tumor shape (**A**, arrow) and a heterogeneous contrast-enhancement of the tumor. Axial T2-weighted MRI illustrates the loss of integrity of the arachnoid layer (**B**, dashed arrow), a regular round tumor shape, and a mild peritumor brain edema (*). The lesion is hyperintense as compared to the brain tissue. Axial T2-weighted MRI shows intratumor calcifications (**C**, arrow) and a clear cerebrospinal fluid cleft at the brain/tumor surface (**C**, dashed arrow), indicating an integer arachnoid layer. Figure d depicts capsular (**D**, arrow) and homogenous contrast-enhancement on coronal T1-weighted MRI.

Finally, correlations between the presence of FTV and characteristics on preoperative radiological imaging were analyzed (Figure 4, Table 3). Disruption of the arachnoid layer (*p* < 0.001) was strongly correlated with FTV. Similarly, FTV tended to occur more often in tumors irregular in shape (*p* = 0.051). In multivariate analyses adjusted for patients’ age, sex, tumor location, histopathological subtype, and all analyzed radiological variables, disruption of the arachnoid layer on preoperative MRI was found to be the only and strong predictor of FTV (OR: 3.99, 95%CI 2.33–6.82; *p* < 0.001).

## 3. Discussion

In the present study, FTV were found in almost 50% of grade I meningiomas and were strongly associated with VD, increased expression of VEGF, PlGF, and IGFBP-3, and a 2-fold increased risk of tumor recurrence.

The WHO classification of brain tumors currently comprises 15 meningioma subtypes, which differ in morphology, recurrence rate, and aggressiveness. Moreover, nine different histological subtypes of grade I meningiomas have been described, reflecting the heterogeneity of this tumor entity [1]. In the present study, a previously undescribed histological feature was detected, consisting of an increased number of fibrotic tumor vessels, observed after EvG-staining and confirmed in the CD34-staining, showing no association with other histological subtypes. Among grade I meningiomas, well-vascularized histological subtypes like angiomatous meningiomas are rare but have been extensively described [18,20,21]. In addition to a high vascular density, they frequently show FTV in their macrovascular subtype. Thus, VD was shown to be distinctly higher in angiomatous tumors than in other grade I meningioma subtypes in our series. However, in contrast to meningiomas displaying FTV, angiomatous meningiomas are usually characterized by low recurrences rates (1.8–2.1% [18,20,21]). Due to the low incidence, in contrast to meningothelial and transitional meningiomas, only four angiomatous meningiomas could be included in our study. Therefore, the morphological distinction between angiomatous meningiomas and meningiomas with FTVs should be further investigated in a larger patient collective.

Tumor vessel fibrosis was independent of most analyzed clinical variables and also similar in primary diagnosed tumors and recurrent lesions. However, univariate and multivariate analyses showed that the risk of recurrence was increased 2-fold in tumors with as compared to meningiomas without FTV. Vascularization is commonly found in rapidly growing tumors and corresponds to a higher metabolic demand of these lesions. Correspondingly, Karsy et al. reported correlations of vascularization and tumor volume in a series of grade I meningiomas [17].

**Figure 5:**
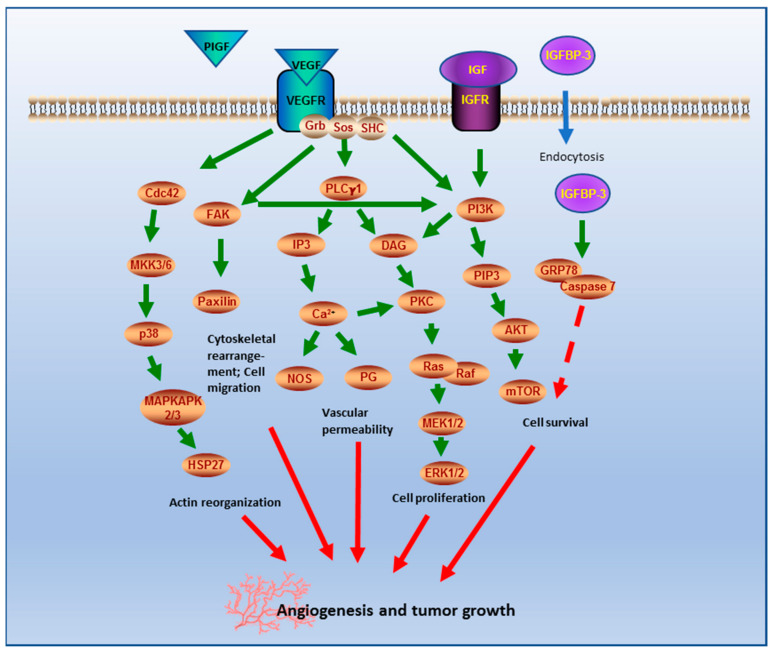
Simplified scheme of putative role, function, and involvement of VEGF, PlGF, and IGFBP-3 in signaling pathways described in angiogenesis and tumor growth. The downstream signaling pathway of the VEGF receptor (VEGFR), including adaptor proteins son of sevenless (SOS), growth receptor bound (Grb) 2, and Src homology 2 domain, can be activated by the ligands VEGF and PlGF. The activation of the mitogen-activated protein kinase (MAPK) signaling pathway via cell division control protein 42 homologous (Cdc42), mitogen-activated protein kinase 3/6 (MKK3/6), p38 mitogen-activated protein kinase (p38/MAPK), and MAP kinase-activated protein kinase 2/3 (MAPKAPK 2/3) triggers actin reorganization via heat shock protein 27 (HSP27), which promotes migration of endothelial cells. The activation of focal adhesion kinase (FAK) results in a cytoskeletal rearrangement by modulating the adhesion protein paxillin. The cleavage of inositol-1,4,5-trisphosphate (IP3) by PLCγ (phospholipases C γ) provokes the release of calcium (Ca^2+^), which contributes to vascular permeability via the release of prostaglandins (PG) and endothelial nitrogen monoxide synthase (NOS). PLCγ also leads via diacylglycerol (DAG) to the activation of the protein kinase C (PKC)-rapidly accelerated fibrosarcoma (Raf)-rat sarcoma (Ras)/mitogen-activated protein kinase (MEK)/extracellular-signal regulated kinases (ERK) pathway, which initiates DNA synthesis to promote endothelial cell proliferation. The phosphoinositide 3-kinase (PI3K)/protein kinase B (Akt)/mammalian target of rapamycin (mTOR) pathway plays a crucial role in cell proliferation and cell survival. PI3K can be activated by VEGFR and by IGFR. There is also a direct stimulation of FAK. IGFBP-3 can bind to 78-kDA glucose-regulated protein (GRP78). This binding can result in proapoptotic and prosurvival activity (based on [23,33,34,38,39,40,41,42,43]).

To evaluate the underlying mechanism of angiogenesis, proteome analyses were performed and revealed increased expression of VEGF, PlGF, and IGFBP-3 in grade I tumors displaying FTV. VEGF stimulates angiogenesis by migration and tube formation of new endothelial cells and promotes angiogenesis in various brain tumors [22,23,24]. In accordance with our results, upregulated VEGF expression has been described in meningiomas with increased vascularity, neoangiogenesis, and peritumoral edema [14,25,26,27]. Furthermore, increased expression of VEGF appears to be associated with tumor size, WHO grade, and recurrence [14,25,26]. In addition, VEGF appears to lead to increased deposition and cross-linking of collagen in the vessels and the tumor matrix, which would explain the morphology of increased fibrosis of the blood vessels in our study [28]. The activation of the PlGF/VEGFR1 axis seems to be important for neo-angiogenesis under pathological conditions and is associated with a reduced survival of patients with colorectal cancer [29,30]. PlGF expression has been demonstrated in smaller series of meningiomas with unclear clinical relevance [25,31,32]. The insulin-like growth factor (IGF) axis is composed of two ligands (IGF-1 and IGF-2) and six binding proteins (IGFBPs), and plays a major role in cell growth and survival. Extracellular IGFBP-3 binds and transports circulating IGFs. Thus, IGFBP-3 influences signaling by increasing or decreasing the access of ligands to receptors [33]. IGFBP-3 has a higher affinity to the IGF receptor (IGFR) than IGF and can therefore reduce the bioavailability of IGF [34]. Binding to the IGFR activates the AKT and ERK1/2 signaling pathways that regulate cell proliferation and cell survival. Overexpression of IGFBP-3 has been described in various tumors and is associated with metastasis and reduced survival [35,36]. Previous studies already reported an upregulation of IGFBP-3 in atypical and anaplastic meningiomas [37]. In summary, the results of our study suggest possible roles of VEGF, PlGF, and IGFBP-3 during tumorigenesis and/or tumor behavior in grade I meningiomas. A hypothetical scheme presenting the putative functions of VEGF, PlGF, and IGFBP-3 in signaling pathways involved in angiogenesis and tumor growth is shown in Figure 5 (based on our own findings and references [23,33,34,38,39,40,41,42,43]).

In this study a homogeneous tumor group has been detected, which is characterized by FTV, increased VD, and upregulation of VEGF, PlGF, and IGFBP-3 expression. To our knowledge, these characteristics have not been commonly associated with grade I meningiomas yet. Hence, the question arises whether the described characteristics are also represented by distinct molecular alterations, such as DNA methylation patterns [44]. The current results also insinuate that a subset of meningiomas might potentially respond to anti-angiogenic therapy. While patients with refractory meningiomas showed a good response to therapy with a VEGF inhibitor in a phase II trial [15], an IGFBP-3 inhibitor has not yet been administered for intracranial tumors. The PlGF inhibitor TB403 is currently being evaluated in a phase I/II clinical trial for the treatment of refractory medulloblastoma (ClinicalTrials.gov Identifier: NCT02748135).

Fibrotic blood vessels can occur in many diseases. Fibrotic diseases have shown the important relationship between angiogenesis, vascular remodeling, and fibrosis [45,46]. The expression of VEGF appears to be essential for the pathogenesis of early vascular and possibly fibrotic changes. Elevated levels of VEGF in patients with diffuse scleroderma were associated with faster and more severe disease progression [47,48]. VEGF was also associated with retinal blood vessel leakage, which is an essential element of diabetic retinopathy [49]. VEGF therefore represents an interesting target for therapeutic intervention in diabetic retinopathy and intravitreal administration of VEGF inhibitors, such as bevacizumab, which has already been used in the treatment of diabetic retinopathy [49,50]. In atherosclerosis, which may also involve fibrotic blood vessels, VEGF has been attributed both pro- and anti-atherosclerotic effects [51,52]. However, the mechanisms of fibrosis in the area of a tumor have not yet been investigated in detail. Increased angiogenesis in tumors is often associated with the formation of immature blood vessels [53]. VEGF has already been described as an important, predominantly hypoxia-induced factor and it is conceivable that VEGF, among other mechanisms, induced the formation of FTVs [53].

Among all analyzed radiological data, FTV was associated with an irregular tumor shape and disruption of the arachnoid layer at the brain/tumor surface. Remarkably, for both characteristics, previous studies have revealed associations with tumor recurrence [54]. Multivariate analyses confirmed disruption of the arachnoid layer as a strong and independent predictor for the presence of FTV. As microscopic analyses were not focused on the brain/tumor surface in the current study series, we cannot provide any histological correlate and a direct association can hardly be hypothesized. Uchida et al. suggested that the T2-hyperintense rim at the brain-meningioma interface reflects a microvascular capsule layer [55]. Hence, it might be possible that the expression of VEGF, PlGF, and IGFBP-3 leads to angiogenesis and promotes increased VD in these tumors.

Although limitations of the study are the retrospective nature and partially semiquantitative analyses (i.e., immunohistochemistry), it involved extensive microscopic and radiological analyses in a large series of grade I meningiomas with detailed follow-up data. The results therefore suggest an important role of angiogenesis in grade I meningiomas and reveal strong correlations with expression of angiogenesis-related proteins, prognosis, and imaging characteristics. Based on the results of the present study, it may be considered whether a statement on the presence of fibrotic blood vessels should be included in the pathology report for further postoperative therapy management. Aside from allowing identification of individuals with increased risk of recurrence after surgery for grade I meningiomas, our data also present potential promising targets (e.g., VEGF, PlGF, and IGFBP-3) for adjuvant antiangiogenic therapy in these patients.

## 4. Material and Methods

### 4.1. Data Collection

Clinical data, radiological imaging, and histopathological diagnosis of all patients who underwent surgery for intracranial grade I meningioma in our department between 1991 and 2015 were reviewed as described previously [56,57,58,59,60,61]. Data included patient sex and age at the time of surgery; the extent of resection, classified as gross total resection (GTR, Simpson grades I–II) and subtotal resection (STR, Simpson grades III–V) [62]. Tumor location was classified as “convexity,” “falx/parasagittal,” “skull base,” “posterior fossa,” or “intraventricular.” Maximum safely achievable tumor resection/reduction was performed in all patients. All samples were neuropathologically examined according to the 2016 WHO Classification of Central Nervous System Tumors ([1] Table 1). Adjuvant irradiation was recommended after subtotal resection of recurrent lesions in concordance with the local tumor board. No chemotherapy was administered. Postoperative care was performed by physical follow-up examinations and gadolinium-enhanced magnet resonance imaging (MRI) at 3 and 6 months after surgery. Imaging was analyzed for tumor progression by a team of at least two independent observers, including one neurosurgeon and one neuroradiologist, and repeated in annual intervals. Progression was defined as any radiologically detectable tumor growth with or without subsequent indication for further treatment. Data collection and scientific use were approved by the local ethics committee (Münster 2007-420-f-S and Münster 2018-061-f-S).

### 4.2. Histopathological Analyses

Hematoxylin and eosin (HE) and Elastica–van Gieson (EvG)-stained tissue sections were independently evaluated by two pathologists in a blinded manner. Fibrotic tumor vessels (FTV) were diagnosed by the presence of collagen-rich, low-cellular lining of the vessels’ walls (Figure 1A). If more than 50% of the blood vessels counted featured fibrotic blood vessels, the specimen was considered positive. In case of discrepant findings, the results were discussed and slides were re-examined together. The blood vessels were visualized by EvG and CD34 staining (CD34; 1:200; Beckman Coulter GmbH, Marseille, France; Figure 1A). For quantification of the median vessel density (VD), the median number of vessels in four consecutive high power fields in the region of interest (ROI) was counted using bright field microscopy (magnification 200×; [63]; Supplement Figure S1). Leptomeningeal vessels were not taken into account. Validation of the histological assessment (intra-observer and inter-observer reproducibility) was assessed using Cohen’s κ statistic. Statistical calculations were performed with version 22 of the package SPSS (SPSS, Chicago, IL, USA).

### 4.3. Proteome Profiling Array

Proteome profiling was performed in a subset of tumors with (*n* = 8) and without FTV (*n* = 7) to analyze expression of putative pro-angiogenic proteins using human angiogenesis arrays (ARY007, R&D Systems, Minneapolis, MN, USA). The sample size for the experimental design was calculated using the free G*power 3.1-software (http://www.gpower.hhu.de). A total of 15 meningiomas (9 meningothelial and 6 transitional) derived from 12 women and three men were included (Supplement Table S1). Selection was performed in a blinded fashion from the collective of all meningiomas. Total protein was isolated from fresh frozen material according to manufacturer’s protocol. Therefore 30–50 mg tissue was homogenized in 1% Triton X-100 in PBS with protease inhibitors; centrifuged supernatants were kept at −80 °C until use. The same amount of protein in each sample group was pooled. A total amount of 250 µg of protein was applied to each respective array membrane from the assay kit for the expression of proteins associated with angiogenesis. The pooled samples were analyzed according to manufacturer’s protocol provided with the assay kit. Expression levels of 55 angiogenesis associated proteins were evaluated by densitometric analyses using the Fusion FX (Vilber, Marne-la-Vallée, France) in combination with the ImageJ software v1.41 (National Institute of Health). For each spot on the membrane, the optical density was measured and the cut off signal level was set to 10% of the respective reference spots. Only regulations of more than 20% were considered as relevant and were further analyzed.

### 4.4. Immunohistochemical Analysis

To verify the results of the proteome profiling, immunohistochemical stainings were performed for vascular endothelial growth factor (VEGF; 1:200; Thermo Fisher, Waltham, MA, USA), placental growth factor (PlGF; 1:200; Proteintech Group Inc., Rosemont, IL, USA), and insulin-like growth factor-binding protein-3 (IGFBP-3; 1:50; Thermo Fisher Scientific, Waltham, MA, USA). Immunohistochemistry was performed using the avidin-biotin-peroxidase technique. Secondary antibodies were biotinylated goat anti-mouse and anti-rabbit immune globulins. Diaminobenzidine (Leica Biosystems Nussloch, Germany) served as chromogen. Placental and renal tissues were used as positive controls. Omission of the primary antibody served as a negative control. Endomembrane expression of VEGF, PlGF, and IGFP-3 was taken into account. Due to the homogeneous endothelial staining and intensity, the expression was evaluated semi-quantitatively in present (positive) and absent (negative) by two observers independently. Representative images of blood vessels without protein expression are shown in the Supplement (Figure S2).

### 4.5. Radiological Imaging

Radiological data had been collected from previous studies ([56,64,65], Figure 4). Briefly, several imaging variables found to be associated with prognosis and/or high-grade histology in a systematic review [54] were analyzed by a team of two independent observers. Tumor and edema volumes (VT and VE) were calculated using the established formula for a spheroid V = 4/3 × π × r1 × r2 × r3, where “r” is the tumor radius at the site of its largest extension in axial (r1), coronal (r2), and sagittal (r3) planes [58]. The integrity of the arachnoid layer was analyzed on T2-weighted MRI and was classified as intact in case of a distinct tumor border and/or evidence of a cerebrospinal fluid cleft at the brain/meningioma surface. Intratumoral contrast enhancement was classified as heterogeneous or homogenous on T1-weighted images. Intensity of the tumor and the presence of intratumoral calcifications were analyzed on T2-weighted MRI and classified as hyper, iso or hypo-intense compared to the grey matter and present or absent, respectively. Enhancement of the tumor capsule was evaluated on gadolinium-enhanced T1-weighted imaging as absent or present. The tumor shape was classified as regular or irregular, e.g., in terms of mushroom-like growth, on T1-weighted contrast-enhanced imaging.

### 4.6. Statistical Analyses

Data are described by absolute and relative frequencies for categorical variables, and median and range for continuous variables. Correlations between categorical and continuous variables were analyzed by Fisher’s exact and unpaired t-tests. Progression free survival (PFS) was defined as the duration between surgery and radiologically confirmed tumor recurrence/progression or, in case of an event-free course, the date of last follow-up. Distribution of PFS was visualized by Kaplan–Meier plots and compared by Log-rank tests. Correlations between the included variables and recurrence were analyzed by Cox regression analyses in univariate and multivariate tests. The results of univariate and multivariate analysis are described with odds (OR) or hazards ratios (HR), 95%-confidence intervals (CI), and backward Wald-test *p*-values. All analyses were performed using SPSS Statistics (Version 22, SPSS, Chicago, IL, USA).

## 5. Conclusions

The results of the present study suggest that angiogenesis not only has an important impact on tumor biology and tumor growth in atypical and anaplastic meningiomas, but also in grade I meningiomas, which are characterized by increased vascular density as well as altered vascular structure, and correlate with angiogenesis-related protein expression, prognosis, and imaging characteristics. Aside from allowing identification of individuals with increased risk of tumor recurrence after surgery for grade I meningiomas, our results also present promising targets for adjuvant antiangiogenic therapy in these patients.

## Figures and Tables

**Table 1:** Baseline clinical and histopathological variables.

Variable	Frequency *n* (%)
Primary diagnosis	275 (93%)
Recurrence	20 (7%)
Age (median, range)	57 years (16–88 years)
Males	77 (26%)
Females	218 (74%)
Extent of resection	
Gross total (GTR, Simpson I/II)	199 (70%)
Subtotal (STR, Simpson III-V)	84 (30%)
Histopathological subtype	
Meningothelial	185 (63%)
Transitional	84 (29%)
Angiomatous	4 (1%)
Fibrous	12 (4%)
Secretory	10 (3%)
Tumor location	
Convexity	93 (32%)
Falx/parasagittal	38 (13%)
Skull base	146 (49%)
Posterior fossa	14 (5%)
Intraventricular	4 (1%)
Tumor/edema volume	
Tumor volume (median, range)	10.00 ccm (0.02–267.77 ccm)
Edema volume (median, range)	0.00 ccm (0.00–364.63 ccm)
Intensity on T2-weighted MRI	
Hypointense	143 (52%)
Isointense	17 (6%)
Hyperintense	114 (42%)
Further radiological criteria	
Arachnoid layer disrupted/absent	154 (52%)
Heterogeneous T1 contrast enhancement	127 (44%)
Tumor shape irregular	108 (38%)
Tumor calcifications	64 (23%)
Capsular contrast enhancement	100 (37%)

Data about the patients’ age, sex, and histopathological subtype were available in all cases; the extent of resection was known for 283 (96%) patients. Regarding radiological imaging, data about tumor location and the arachnoid layer were available for all, and data about tumor and edema volumes, tumor intensity on T2-weighted MRI, heterogeneity of contrast-enhancement, tumor shape, calcifications, and capsular contrast-enhancement were available for 269 (91%), 265 (90%), 274 (93%), 286 (97%), 282 (96%), 276 (94%), and 268 patients (91%), respectively.

**Table 2:** Correlations between clinical and histopathological variables and fibrotic tumor vessels (FTV).

Variable	Frequency *n*(%)		
Indication for Surgery	non-FTV	FTV	*p*-Value
Primary diagnosis	144 (49%)	131 (44%)	0.503
Recurrence	11 (4%)	9 (3%)	
Age (mean, range)	58 years (16–88 years)	55 years (16–88 years)	0.065
Sex			0.288
Male	36 (12%)	41 (14%)	
Female	119 (40%)	99 (34%)	
Extent of resection			
Gross total (GTR, Simpson I/II)	110 (39%)	89 (31%)	0.136
Subtotal (STR, Simpson III/IV)	37 (13%)	47 (17%)	
Histopathological subtype			0.136
Meningothelial	92 (31%)	93 (32%)	
Transitional	50 (17%)	34 (12%)	
Angiomatous	0 (0%)	4 (1%)	
Fibrous	7 (2%)	5 (2%)	
Secretory	6 (2%)	4 (1%)	
Tumor location			0.493
Convexity	50 (17%)	43 (15%)	
Falx/parasagittal	22 (8%)	16 (5%)	
Skull base	71 (24%)	75 (25%)	
Posterior fossa	10 (3%)	4 (1%)	
Intraventricular	2 (1%)	2 (1%)	
Angiogenic proteins			
IGFBP-3 expression/absent	68 (24%)/82 (28%)	109 (38%)/29 (10%)	<0.001
PIGF expression/absent	52 (18%)/99 (34%)	84 (29%)/55 (19%)	<0.001
VEGF expression/absent	64 (22%)/88 (30%)	87 (30%)/52 (18%)	<0.001
Vessel density (vessels/4 HPF)	35.8 ± 28.0	72.2 ± 42.8	<0.001

**Table 3:** Correlations between the occurrence of fibrotic tumor vessels (FTV) and radiological variables.

Variable	Frequency *n* (%)		
	non-FTV	FTV	*p*-Value
Tumor/edema volume			
Tumor volume (median, range)	10.36 ccm (0.02–141.91 ccm)	9.97 ccm (0.21–267.77 ccm)	0.108
Edema volume (median, range)	0.00 ccm (0.00–364.63)	0.47 ccm (0.00–355.80)	0.621
Intensity on T2-weighted MRI			0.346
Hypointense	72 (26%)	71 (26%)	
Isointense	9 (3%)	8 (3%)	
Hyperintense	59 (22%)	55 (20%)	
Further radiological criteria			
Arachnoid layer disrupted/absent	56 (19%)/99 (34%)	98 (33%)/42 (14%)	<0.001
Heterogeneous T1 contrast enhancement	62 (22%)	65 (23%)	0.429
Tumor shape irregular	47 (17%)	61 (22%)	0.051
Tumor calcifications	35 (13%)	29 (11%)	0.480
Capsular contrast enhancement	58 (22%)	42 (16%)	0.077

## Supplementary Materials

The following are available online at https://www.mdpi.com/2072-6694/12/10/3075/s1. Figure S1. Quantification of blood vessels using EvG (mean 53.8 ± 39.5 vessels/4 HPF) and CD34 staining (mean 52.8 ± 39.8 vessels/4 HPF). Figure S2. Representative images of blood vessels (arrow) without VEGF (A), IGFBP-3 (B), and PlGF (C) expression (original magnification 200×). Table S1. Clinical and histopathological variables of patients undergoing proteome analysis. n = number, men. = meningothelial, tran. = transitional, f = female, m = male, PFS = progressionfree survival, OS = overall survival, HPF = high powerfield.
