# Supplementary material for: Occurrence of Fibrotic Tumor Vessels in Grade I Meningiomas Is Strongly Associated with Vessel Density, Expression of VEGF, PlGF, IGFBP-3 and Tumor Recurrence
Source: Cancers (Basel). 2020 Oct 21;12(10):3075. doi: 10.3390/cancers12103075 (PMC7593950; doi:10.3390/cancers12103075)
Supplement: Supplementary file 1 [file cancers-12-03075-s001.zip › cancers-970344-supplementary.docx]

Supplementary Material: Occurrence of Fibrotic Tumor Vessels in Grade I Meningiomas is Strongly Associated with Vessel Density, Expression of VEGF, PlGF, IGFBP-3 and Tumor Recurrence

Katharina Hess, Dorothee Cäcilia Spille, Alborz Adeli, Peter B. Sporns, Karina Zitta,
Lars Hummitzsch, Julian Pfarr, Walter Stummer, Benjamin Brokinkel and Rouven Berndt
and Martin Albrecht

**Figure S1.**  Quantification of blood vessels using EvG (mean 53.8 ± 39.5 vessels/4 HPF) and CD34 staining (mean 52.8 ± 39.8 vessels/4 HPF).


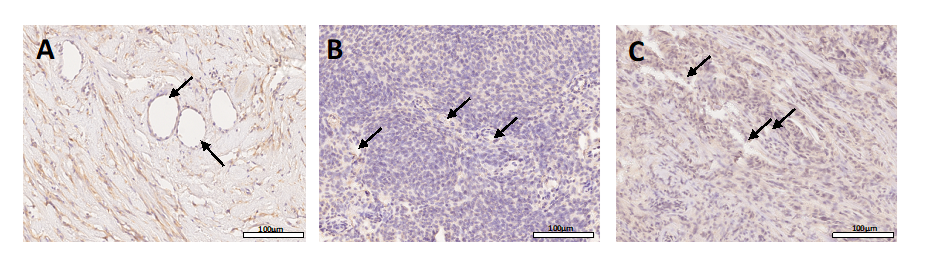


**Figure S2.** Representative images of blood vessels (arrow) without VEGF (**A**), IGFBP-3 (**B**) and PlGF (**C**) expression (original magnification 200×).

**Table S1.** Clinical and histopathological variables of patients undergoing proteome analysis

| ***n*** | **Histological**  **Subtype** | **Age** | **Sex** | **PFS** | **OS** | **Vessel/4HPF** | **IGFBP3** | **PlGF** | **VEGF** | **FTV(%)** |
| --- | --- | --- | --- | --- | --- | --- | --- | --- | --- | --- |
| 1 | men. | 72 | f | 18 | 74 | 104 | yes | yes | yes | 94 |
| 2 | men. | 42 | f | 0 | 0 | 20 | yes | yes | yes | 90 |
| 3 | tran. | 85 | f | 70 | 70 | 44 | yes | no | yes | 84 |
| 4 | men. | 34 | f | 8 | 78 | 55 | yes | no | no | 89 |
| 5 | men. | 53 | m | 11 | 11 | 43 | yes | yes | yes | 88 |
| 6 | tran. | 58 | f | 109 | 109 | 71 | yes | no | no | 93 |
| 7 | men. | 62 | f | 28 | 88 | 31 | yes | yes | yes | 68 |
| 8 | tran. | 65 | f | 64 | 64 | 40 | yes | yes | yes | 93 |
| 9 | men. | 59 | f | 33 | 33 | 83 | no | no | no | 82 |
| 10 | tran. | 74 | f | 71 | 71 | 12 | no | no | no | 8 |
| 11 | men. | 70 | f | 66 | 66 | 148 | yes | yes | yes | 26 |
| 12 | men. | 36 | m | 150 | 150 | 16 | no | yes | yes | 19 |
| 13 | men. | 28 | f | 70 | 94 | 82 | no | no | no | 20 |
| 14 | tran. | 75 | f | 86 | 86 | 19 | no | no | no | 11 |
| 15 | tran. | 60 | m | 53 | 53 | 24 | no | yes | yes | 17 |

*n* = number, men. = meningothelial, tran. = transitional, f = female, m = male, PFS = progressionfree survival, OS = overall survival, HPF = high powerfield.

| 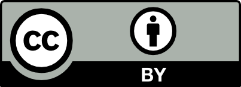 | © 2020 by the authors. Licensee MDPI, Basel, Switzerland. This article is an open access article distributed under the terms and conditions of the Creative Commons Attribution (CC BY) license (http://creativecommons.org/licenses/by/4.0/). |
| --- | --- |
